# Study on Major Diseases, Pests, and Predators of Honeybees (*Apis mellifera* L.) in East Shewa and West Arsi Zones of Oromia, Ethiopia

**DOI:** 10.1155/vmi/6662250

**Published:** 2026-05-30

**Authors:** Taye Beyene, Mekonen Woldetsadik, Desta Abi, Sisay Eshetu

**Affiliations:** ^1^ Department of Biology, Adami Tulu Agricultural Research Center, P.O. Box 35, Batu, Oromia, Ethiopia

**Keywords:** diseases, honeybee, pests, predators, prevalence, Varroa mites

## Abstract

A cross‐sectional study design was conducted across agroecological zones in East Shewa and West Arsi Zones of Oromia, Ethiopia, from September 2020 to November 2021 to identify the types, prevalence, and potential risk factors associated with honeybee diseases, pests, and predators. Purposive sampling involved interviewing 115 households and analyzing 80 samples via laboratory diagnosis. Data were coded and stored in Microsoft Excel spreadsheets and then analyzed using descriptive statistics, frequency distributions, a rank index formula, and chi‐square (*χ* ^2^) tests in SPSS Version 16. The results revealed that ants were the most common pests observed in honeybee colonies (27.4%), followed by wax moths (21.9%), honey badgers (14.4%), bee‐eater birds (10.8%), small hive beetles (8.9%), lizards (6.6%), spiders (4.9%), death’s head hawkmoths (3.2%), and wasps (1.6%). Annual absconding rates reached 60.4% in Dodola, 64.5% in Wando, 70.8% in Adami Tulu, and 84% in Dugda. Laboratory analyses confirmed high positivity for amoebiasis (82.5%), varroosis (83.8%), nosemosis (43.8%), small hive beetles (33.8%), and bee lice (33.8%), with no detections of American foulbrood, European foulbrood, chalkbrood, stonebrood, and tracheal mites. These threats severely impact honey production, emphasizing the need for beekeeper vigilance through effective control measures and seasonal colony management to bolster hive resilience.

## 1. Introduction

Ethiopia is noticeable for its diversified agroecology and biodiversity, which makes the country ideal for the existence of a huge number of honeybee colonies [1]. Ethiopia, with the highest number of bee colonies and surplus honey flora, leads Africa in honey and beeswax production. These have enabled Ethiopia to take a total share of honey production of around 23.58% and 2.13% of the African and world totals, respectively [2].

Beekeeping offers multifaceted benefits, enhancing food and cash crop productivity while aiding natural resource conservation through pollination [3, 4]. Besides this, beekeeping serves as a vital income source for landless people in harsh environments, while also aiding poverty reduction and generating foreign currency through honeybee product sales. It provides accessible livelihoods without requiring large land holdings, enabling sustenance in challenging terrains. However, sector productivity is declining due to falling honeybee populations, driven by extensive agricultural chemical use and the emergence of pests, predators, and diseases [5, 6].

Honeybee diseases, pests, and predators directly cause bee deaths, reducing honeybee products and harming domestic and international marketing efforts. These threats lead to colony losses, diminished yields of honey and other products, and weakened market competitiveness [7]. Ethiopia’s agroecological conditions favor not only honeybees but also various diseases, pests, and predators that interact with and threaten honeybee colonies [8].

In Ethiopia, protozoa (*Nosema apis* and *Amoeba*), fungi (chalkbrood disease), parasitic mites (*Varroa*), small and large hive beetles, wax moths, ants, wasps, bee‐eater birds, and honey badgers were identified as major honeybee diseases and pests [9–11]. However, evidence on the magnitude and distribution of honeybee pests and diseases remains underexplored, with severity varying across colonies, apiaries, regions, and weather conditions [12]. On the other hand, bacterial and viral diseases, highly contagious threats, lack documentation in the country [13]. Limited knowledge exists on the status, prevalence, distribution, and economic impacts of honeybee diseases and pests in East Shewa and West Arsi Zones of Oromia, including unreported diseases. Highly contagious bacterial and viral diseases remain undocumented in Ethiopia, complicating control efforts. This study identifies their occurrence, prevalence, associated risk factors, and distribution to enable early intervention and support sustainable beekeeping.

## 2. Materials and Methods

### 2.1. Study Area

**FIGURE 1:**
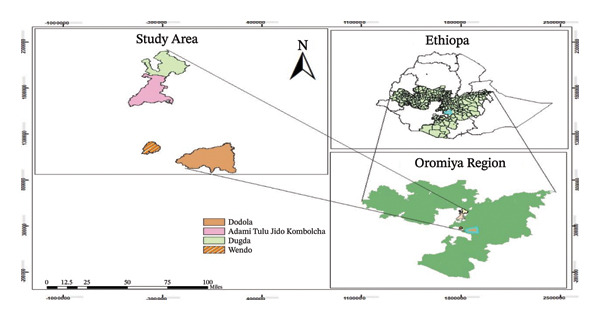
Map of study areas.

The diagnostic study was conducted in the selected four districts: Dugda and Adami Tulu Jido Kombolcha districts from East Shewa Zone and Dodola and Wando districts from West Arsi Zone of Oromia. Dodola district represented highlands; Wando district represented midlands; whereas Dugda and Adami Tulu Jido Kombolcha districts represented lowland agroecology. Dugda district is situated between 8°02′59″N latitude and 38°43′59″E longitude with altitude ranges from 1576 to 1750 m above sea level (Figure 1). The mean annual rainfall of the district is 795.4 mm, and the minimum and maximum temperatures range from 13.6°C to 29.2°C. Adami Tulu Jido Kombolcha is situated between 7°9′N latitude and 38°7′E longitude, and the altitude is 1650 m above sea level (Figure 1). The mean annual rainfall is 760 mm, and the minimum and maximum temperatures range from 12.6°C to 27°C [14]. Wando district is characterized by midland agroecological zone. It is situated between 07°05′35″N latitude and 038°36′66″E longitude. The altitude of this district ranges from 1700 to 2300 m above sea level (Figure 1). The annual average temperature ranges from 17°C to 19°C. Dodola district is situated between 06°59′N latitude and 39°11′E longitude with an altitude of 2362–2493 m above sea level (Figure 1). The mean annual rainfall ranges from 800 to 1200 mm, and the minimum and maximum temperature ranges from 13°C to 26°C.

### 2.2. Sampling Techniques and Sample Size Determination

Before the actual survey was conducted, secondary data and baseline information were collected by consulting responsible extension officers at zonal and district levels. Then, semistructured questionnaires were prepared and pretested. Two administrative zones, four districts, and 12 rural kebeles representing highland, midland, and lowland agroecologies were sampled using purposive sampling. A total of 12 kebeles, three kebeles in each district, were selected at 10‐km intervals between the adjacent sampling kebeles. Respondent beekeepers were randomly selected using Yamane’s sample size determination method.

(1) n=N1+Ne2,

where SS = required sample size; *N* = total population; *e* = margin of error (10%). Accordingly, a total of 115 respondents were sampled from 12 selected kebeles (PAs). Field observations assessed pests and predators’ presence, with beekeepers queried on disease occurrences and their reports documented. The sample size of inspected colonies was determined based on sample size determination in the random sampling method for an infinite population, using 50% of the expected prevalence of bee diseases and pests and a 95% confidence interval at 5% absolute precision.

(2) n=1.962·Pexp1−Pexpd2,

where *n* is the required sample size; Pexp is the expected prevalence (50%); *d* ^2^ is the precision (5%).

### 2.3. Study Design

A cross‐sectional study was conducted from September 2020 to November 2021 in the selected districts of East Shewa and West Arsi Zones of Oromia to assess the prevalence of common honeybee diseases, pests, and predators in the field through inspection and examination of samples at the laboratory using their respective protocols. A questionnaire survey was carried out during the diagnostic study to determine the honey production system and constraints caused by pests and predators. The prevalence for apiary and colony levels was calculated following the protocols in [15]:

(3) prevalence=number of positive casestotal number of sampled populations×100.

### 2.4. Study Methodology

A single beehive was considered as one sample unit. Types of hives and agroecology were considered as explanatory variables (risk factors) and examined whether they have an impact on the occurrence of honeybee diseases and pests. Honeybee hives were categorized as modern and transitional hives. Three altitude categories were considered: highland (> 2400 m), midland (1800–2400 m), and lowland (1800 m) above sea level.

### 2.5. Laboratory Examinations

#### 2.5.1. Nosemosis and Amoebiasis

The laboratory examination of *N. apis* and Amoeba infestation was undertaken using the following procedures. The samples of 20–30 adult honeybees were collected from the hive entrance [16]. The sample bees were collected and preserved in 70% alcohol until laboratory analysis. The abdomen of honeybees from each sample was cut and ground in a mortar containing 5–10 mL distilled water. The mortar and pestle were thoroughly cleaned before being used again. A loop of suspension was placed on the microscopic slide using a sterilized loop and covered with a cover slide. The suspension was examined under a light microscope using 40 magnification power for the presence of Nosema spores and Amoeba cysts.

#### 2.5.2. Chalkbrood

The chalkbrood disease mummies were checked at the bottom board of the hive entrance, in the comb cells, and on the ground beneath the hive entrance. Mummies were moistened with distilled water, and the supernatants were placedon microscope slides, covered with coverslips, and examined under 40X magnification for spores, spore balls, and cysts of *Ascosphaera apis* [17].

#### 2.5.3. American Foulbrood (AFB) and European Foulbrood (EFB)

Field diagnostic procedures for AFB and EFB were used based on the procedure in [17]. To test the AFB and EFB diseases, larvae that had discolored, exhibited a melted appearance, ropiness, hard and dark scales that adhere strongly to the lower sides of the cell, and protruding tongue were checked for their presence. The brood showing one of the above clinical symptoms, brood samples were prepared on microscopic slides for further laboratory test following the method described in [17]. Samples were examined using an oil‐immersed Zeiss Axio Vert A.1 light microscope for *Paenibacillus larvae* (in AFB‐positive samples) and *Melissococcus pluton* (in EFB‐positive samples) at 100X magnification.

#### 2.5.4. Varroosis (Varroa Destructor)

Varroa destructor was subjected to laboratory examination, followed by the standard methods for Varroa detection [18]. Adult honeybees were brushed off from the brood comb and directly into a wide‐mouthed plastic container. The collected adult bees were killed using 70% ethyl alcohol and placed in 10 mL of a 1% detergent–water solution (10 mL detergent in 1000 mL water) and vigorously shaken for 1 min to dislodge mites. The mites were collected by filtering the solution through a ladle (8–12 mesh) that held the bees back and let out the mites with the solution. Then, wire gauze was used to hold the mites back and discharge the solution. The wire gauze was turned down to white paper on which the presence/absence of the mite was examined and counted. For brood examinations, samples of a 5 × 5 cm^2^ brood comb area from drone and/or worker pupae broods were taken. About 100 pupae were randomly removed from their cells using forceps and examined for the presence of Varroa mites on workers and drone pupae. The number of Varroa mites observed in both diagnoses (adult and brood) was recorded.

#### 2.5.5. Bee Lice

Adult worker bees from colonies in the study areas were examined for bee louse (*Braula coeca*) using the same procedures used for Varroa mite detection.

#### 2.5.6. Trachea Mites

Samples of 20–30 adult honeybees were collected randomly [16]. The collected samples were preserved in 70% alcohol. Then, the head and the first pair of legs were removed using forceps and scissors. Transverse sections of thoracic discs were sliced and placed directly in a small dish containing 10% potassium hydroxide (KOH). The sliced thoracic disks in KOH were heated and stirred gently near to the boiling point for approximately 10 min until the soft internal tissues dissolved to expAose trachea rings. After being filtered and then cleaned with tap water, the trachea ring fragments were obtained. The disk–trachea suspension was examined for infested parts and *Acarapis woodi* under a dissecting microscope at a 10‐magnification power.

#### 2.5.7. Diagnosis of Major Honeybee Pests and Predators

Investigators were able to identify major honeybee pests and predators through semistructured questionnaires, interviews with beekeepers, and internal and external hive inspection in all the study areas. Because the larvae of the small hive beetle larvae (*Aethina tumida*) had three pairs of anterior prolegs and two pairs of conspicuous brownish dorsal spines on each segment, the infestation of SHB was detected using methods for examining the adult, larval, pupal, and colony stages [19]. Wax moth larvae lack spines but do have many setae (hairs) on each segment with 8 pairs of prolegs (3 pairs, 4 pairs, and 1 pair on the anterior, abdominal, and last segments, respectively) [20]. It creates silken galleries, as opposed to small hive beetles, and was identified through its adult, larval, or pupal colony examination methods as described in [20]. Larvae of small hive beetles have pairs of prominent brownish dorsal spines on each segment with 3 pairs of anterior prolegs only.

Wax moth larvae lack spines but have several setae (hairs) in each segment, with eight pairs of prolegs (three pairs in the anterior, four pairs in the abdominal, and one pair in the final segments). Unlike small hive beetles, wax moths produce silken galleries.

### 2.6. Data Management and Statistical Analysis

The obtained data were coded, tabulated, analyzed, and interpreted using descriptive statistics of SPSS Version 20. Descriptive statistics, such as means, standard deviation, frequency distribution, range, and percentage, are presented in the form of tables. Data were collected, and analysis was done using descriptive statistics and the rank index formula as described in [21]. The chi‐square (*χ* ^2^) test was used to assess the association of the risk factors with the prevalence of the disease and pests. The differences were considered significant at (*p* < 0.05) with a 95% confidence level.

## 3. Results

### 3.1. Demographic Characteristics of the Respondents

From the total of 115 sample households interviewed, 90.4% were male‐headed, and 9.6% were female‐headed (Table 1). The study showed that very limited numbers of female were engaged in beekeeping activities. Of the total beekeepers interviewed, 24.3% were illiterate, 27.8% could read and write, 20.9% attended primary education, 16.5% attended junior education, 8.7% attended secondary education, and 1.7% had a college diploma (Table 1). The respondents’ ages ranged from below 20 years (5.2%), 20–40 years (30.4%), 41–60 years (46.1%), to above 60 years (18.3%). This distribution indicates that most participants were in their productive prime, actively engaged in beekeeping. Beekeeping experience varied, with 9.6% having 1–5 years, 17.4% with 6–10 years, 33% possessing 11–15 years, and 40% exceeding 15 years (Table 1). Overall, the sample reflects seasoned practitioners capable of providing reliable insights into the activity.

**TABLE 1:** Sociodemographic characteristics of the sample respondents (*N* = 115).

Demographic variables	Category	Frequency	Percentage (%)
Sex of respondent	Male	104	90.4
	Female	11	9.6

Educational status of the respondents	Illiterate	28	24.3
	Read and write	32	27.8
	Primary	24	20.9
	Junior	19	16.5
	Secondary	10	8.7
	College diploma	2	1.7

Age of the respondent	< 20	6	5.2
	20–40	35	30.4
	41–60	53	46.1
	> 60	21	18.3

Beekeeping experience	1–5 years	11	9.6
	6–10 years	20	17.4
	11–15 years	38	33
	> 15 years	46	40

### 3.2. Beekeepers’ Perception on Trends of Honey Production and Honeybee Colony

The majority of beekeepers (62.6%, 54.8%) responded that the trend of honey production and honeybee colony had decreased, respectively, and some beekeepers also responded that honey production and honeybee colony are on an increasing trend by 27% and 29.6%, respectively (Table 2). On the other hand, about 10.4% and 15.7% of the sample respondents stated that no change occurred in their honey production and honeybee colonies, respectively (Table 2). The major causes for the decrease in the number of honeybee colonies and productivity of honeybee colonies as mentioned by the respondents were as follows: unwise application of agrochemicals, shortage of bee forage, prevalence of honeybee pests and predators, drought, and deforestation as the main threatening factors for colony population and honey yield decline in the study areas.

**TABLE 2:** Beekeepers perception on trends of honey production and honeybee colony.

Trends	Honeybee colony		Honey production	
	Frequency	Percentage (%)	Frequency	Percentage (%)
Decrease	72	62.6	63	54.8
Increase	31	27	34	29.6
No change	12	10.4	18	15.7

### 3.3. Major Beekeeping Constraints

Beekeepers identified various challenges impacting the beekeeping subsector in their areas. The primary constraints on beekeeping development, ranked in decreasing order, included lack of bee forage, agrochemical applications, pests and predators, drought and deforestation, honeybee diseases, absconding, high costs of improved beehives, and shortages of honeybee colonies (Table 3). These issues highlight the need for targeted interventions to enhance forage availability and pest management.

**TABLE 3:** Major beekeeping constraints in the sample districts.

Constraints	The relative degree of importance according to the respondents								
	1st	2nd	3rd	4th	5th	6th	7th	Index	Rank
Lack of bee forage	55	23	8	3	1	2	0	0.149	1st
Application of agrochemicals	37	20	16	17	8	0	0	0.142	2nd
Pests and predators	35	32	17	0	0	7	4	0.139	3rd
Drought and deforestation	6	46	21	8	5	0	0	0.121	4th
Honeybee diseases	4	38	31	6	8	0	0	0.118	5th
Absconding	9	33	16	14	18	0	0	0.116	6th
High cost of improved beehives	5	40	20	8	4	1	0	0.108	7th
Shortage of honeybee colony	0	37	16	12	15	6	0	0.105	8th

*Note:* Index = sum of (7 ∗ ranked 1st + 6 ∗ ranked 2nd + 5 ∗ ranked 3rd + 4 ∗ ranked 4th + 3 ∗ ranked 5th + 2 ∗ ranked 6th + 1 ∗ ranked 7th) for individual reasons divided by the sum of (7 ∗ ranked 1st + 6 ∗ ranked 2nd + 5 ∗ ranked 3rd + 4 ∗ ranked 4th + 3 ∗ ranked 5th + 2 ∗ ranked 6th + 1 ∗ ranked 7th) for overall constraints.

### 3.4. Major Honeybee Pests and Predators

**FIGURE 2:**
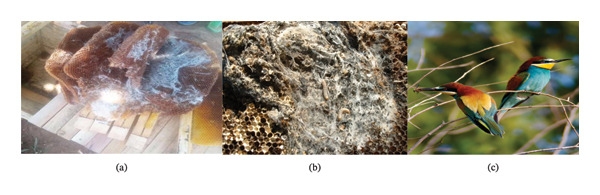
(a, b) Combs affected by wax moths; (c) bee‐eater birds.

Beekeeping production systems in the study areas encounter numerous challenges, with pests and predators identified as primary constraints. Survey results identified ants, wax moth presented in Figure 2, honey badger, bee‐eater birds presented in Figure 2, small hive beetles, lizards, spiders, death’s head hawkmoth, and wasps as primary honeybee pests and predators, ranked by decreasing prevalence (Table 4).

**TABLE 4:** Major honeybee pests and predators in the sampled districts.

Honeybee pests and predators	Relative degree of importance according to the respondents								
	1st	2nd	3rd	4th	5th	6th	7th	Index	Rank
Ants	40	31	17	7	3	0	0	0.274	1st
Wax moths	38	21	9	6	1	0	0	0.219	2nd
Honey badgers	14	17	12	8	2	3	0	0.144	3rd
Bee‐eater birds	9	7	17	4	5	3	1	0.108	4th
Small hive beetles	0	5	15	12	7	8	0	0.089	5th
Lizards	0	10	7	5	8	2	1	0.068	6th
Spiders	5	2	9	1	3	0	0	0.049	7th
Death’s head moths	0	4	6	1	2	0	3	0.032	8th
Wasps	0	2	4	0	0	1	0	0.016	9th

*Note:* Index sum of (7 ∗ ranked 1st + 6 ∗ ranked 2nd + 5 ∗ ranked 3rd + 4 ∗ ranked 4th + 3 ∗ ranked 5th + 2 ∗ ranked 6th + 1 ∗ ranked 7th) for Individual pests and predators divided by the sum of (7 ∗ ranked 1st + 6 ∗ ranked 2nd + 5 ∗ ranked 3rd + 4 ∗ ranked 4th + 3 ∗ ranked 5th + 2 ∗ ranked 6th + 1 ∗ ranked 7th) for overall pests and predators.

### 3.5. Effect of Pests and Predators on Honeybee Colony

Respondents identified ants (both black and red) and wax moths as primary causes of absconding in 80.9% and 74.8% of honeybee colonies, respectively. Beekeepers’ responses further highlighted wax moths (78.3%) and ants (75.7%) as leading factors in honey losses (Table 5). These patterns emphasize the critical role of these pests in reducing honey yields and colony viability. Among the 898 honeybee colonies surveyed, 70.3% (631 colonies) absconded due to pests and predators, including ants, wax moths, honey badgers, bee‐eater birds, and small hive beetles (Table 6). This absconding led to considerable losses of honey and other hive products. According to the respondents’ scores, ants accounted for 18.6% (167 colonies) of absconding cases, wax moths for 18% (162), bee‐eater birds for 14.3% (128), honey badgers for 11.2% (101), and small hive beetles for 8.1% (73). Collectively, these pests and predators contributed substantially to the abandonment of colonies and the associated loss of hive products.

**TABLE 5:** Effect of some pests and predators on honeybee colony at different districts.

Pests and predators	Number of colonies dwindled	Honey losses	Number of colonies absconded
	*N* (%)	*N* (%)	*N* (%)
Ants	81 (70.4%)	87 (75.7%)	93 (80.9%)
Wax moth	84 (73%)	90 (78.3%)	86 (74.8%)
Honey badgers	—	72 (62.6%)	67 (58.3%)
Bee‐eater birds	73 (63.5%)	67 (58.3%)	34 (29.6%)
Small hive beetles	65 (56.5%)	64 (55.7%)	70 (60.9%)
Lizard	35 (30.4%)	52 (45.2%)	—

**TABLE 6:** Number of colonies absconded due to pests and predators in sampled districts.

District	Total colonies	Ants	Wax moth	Honey badger	Bee‐eater birds	Small hive beetles	Absconded
Dodola	321	54 (16.8%)	46 (14.3%)	40 (12.5%)	31 (9.7%)	23 (7.2%)	194 (60.4%)
Wando	228	40 (17.5%)	37 (16.2%)	33 (14.5%)	22 (9.6%)	15 (6.6%)	147 (64.5%)
Adami Tulu	24	5 (20.8%)	7 (29.2%)	—	5 (20.8%)	—	17 (70.8%)
Dugda	325	68 (20.9%)	72 (22.2%)	55 (16.9%)	43 (13.2%)	35 (10.8%)	273 (84.0%)
Total	898	167 (18.6%)	162 (18.0%)	128 (14.3%)	101 (11.2%)	73 (8.1%)	631 (70.3%)

### 3.6. Laboratory Examination of Honeybee Diseases and Pests

#### 3.6.1. Prevalence of *N. apis* and Associated Risk Factors

The overall prevalence of Nosemosis (*N. apis*) was 60% across the study areas (Table 7). Prevalence varied by agroecology, reaching 76.9% in highlands, 54.2% in midlands, and 50% in lowlands, though no statistically significant difference existed among these zones (*p* > 0.05). Modern hives showed the highest *N. apis* prevalence at 83.3%, compared to 25% in transitional hives, with a significant difference between hive types (*p* < 0.05).

**TABLE 7:** Prevalence of *Nosema apis* and associated risk factors.

Variables	Category	No. of hives examined	Prevalence (%)	*χ* ^2^	*p* value
Agroecology	Highland	26	20 (76.9%)	4.693	0.096
	Midland	24	13 (54.2%)		
	Lowland	30	15 (50%)		

Hive types	Modern	48	40 (83.3%)	27.22	0.001
	Transitional	32	8 (25%)		

Total		80	48 (60%)		

#### 3.6.2. Prevalence of Amoeba (*Malpighamoeba mellificae*) and Associated Risk Factors

The overall prevalence of *M. mellificae* was 83.8% in this study (Table 8). Prevalence was highest in highland agroecology at 92.35%, followed by midland (83.3%) and lowland (76.7%), with a statistically significant difference among these zones (*p* < 0.05). Transitional hives showed higher *M. mellificae* prevalence at 87.5% compared to 81.3% in modern hives, with a significant variation between hive types (*p* < 0.05).

**TABLE 8:** Prevalence of amoeba (*M. mellificae*) and associated risk factors.

Variables	Category	No of hive examined	Prevalence (%)	*χ* ^2^	*p* value
Agroecology	Highland	26	24 (92.35%)	2.508	0.025
	Midland	24	20 (83.3%)		
	Lowland	30	23 (76.7%)		

Hive types	Modern	48	39 (81.3%)	0.551	0.418
	Transitional	32	28 (87.5%)		

Total		80	67 (83.8%)		

#### 3.6.3. Prevalence of Varroosis (*Varroa destructor*) and Associated Risk Factors

The overall prevalence of varroosis was 82.5% based on laboratory diagnosis (Table 9). Prevalence was highest in lowland agroecology at 86.7%, followed by midland (83.3%) and highland (76.9%), with a statistically significant difference among these zones (*p* < 0.05). Transitional hives showed the highest varroosis at 93.8%, compared to 79.2% in modern hives, indicating a significant difference between hive types (*p* < 0.05).

**TABLE 9:** The prevalence of Varroa and its associated risk factors.

Variables	Category	No. of hives examined	Prevalence (%)	*χ* ^2^	*p* value
Agroecology	Highland	26	20 (76.9%)	4.0093	0.038
	Midland	24	20 (83.3%)		
	Lowland	30	26 (86.7%)		

Hive types	Modern	48	38 (79.2%)	2.438	0.118
	Transitional	32	30 (93.8%)		

Total		80	66 (82.5%)		

#### 3.6.4. Prevalence of Small Hive Beetle (*A. tumida*) and Associated Risk Factors

The small hive beetle exhibited an overall prevalence of 43.8% across surveyed colonies (Table 10). Transitional hives showed the highest infestation rate at 65.6%, followed by modern hives at 29.2%, with a significant difference between hive types (*p* < 0.05). No significant variation occurred across agroecology (*p* > 0.05), although prevalence reached 28.9% in highlands, 45.8% in midlands, and 56.7% in lowlands.

**TABLE 10:** The prevalence of small hive beetles and its associated risk factors.

Variables	Category	No. of hives examined	Prevalence (%)	*χ* ^2^	*p* value
Agroecology	Highland	26	7 (26.9%)	5.0678	0.079
	Midland	24	11 (45.8%)		
	Lowland	30	17 (56.7%)		

Hive types	Modern	48	14 (29.2%)	10.3704	0.001
	Transitional	32	21 (65.6%)		

Total		80	35 (43.8%)		

#### 3.6.5. Prevalence of Bee Lice (*B. coeca*) and Associated Risk Factors

The overall prevalence of bee lice was 33.8% among 80 examined colonies, as confirmed by laboratory diagnosis (Table 11). Prevalence was highest in lowland agroecology at 53.8%, followed by midland (37.5%) and highland (13.3%), with a statistically significant difference among these zones (*p* < 0.05). Modern hives recorded the highest bee lice infestation at 35.4%, followed by transitional hives at 31.3% (Table 11).

**TABLE 11:** Prevalence of bee lice (*Braula coeca*) and associated risk factors.

Variables	Category	No. of examined	Prevalence (%)	*χ* ^2^	*p* value
Agroecology	Highland	26	4 (13.3%)	10.439	0.005
	Midland	24	9 (37.5%)		
	Lowland	30	14 (53.8%)		

Hive types	Modern	48	17 (35.4%)	0.149	0.699
	Transitional	32	10 (31.3%)		

Total		80	27 (33.8%)		

## 4. Discussion

Of the total 115 sampled households interviewed, 90.4% were male‐headed, while only 9.6% were female‐headed. This distribution shows beekeeping dominated by male‐headed households, with limited female involvement. Such patterns align with reports noting beekeeping as men’s work in Ethiopia [22]. The forest‐based beekeeping system in the area culturally restricts women, as it requires climbing trees to bait swarms or manage hives suspended from branches. These traditions limit women’s access to the practice. Respondents’ age groups among beekeepers and nonbeekeepers ranged from below 20 years (5.2%), 20–40 years (30.4%), 41–60 years (46.1%), to above 60 years (18.3%). These spread highlights a concentration in middle adulthood, aligning with established views that the 15–60 age bracket represents the economically active population [23, 24]. Beekeeping in the study areas involves both literate and illiterate participants. Educational levels among farming households significantly influence development types, lifestyles, and extension service strategies. Among sampled respondents, 24.3% were illiterate, 27.8% could read and write, 20.9% had primary education, 16.5% had junior education, 8.7% had secondary education, and 1.7% held college diplomas. This result is higher than the findings of the author in [25], who reported that 15.1% of the respondents had not received any education in Bure district of Amhara Region. Major constraints hindering beekeeping development in the study areas, ranked in decreasing order, include shortage of bee forage, improper agrochemical application, pests and predators, drought and deforestation, diseases, absconding, high costs of improved hives and accessories, and shortages of honeybee colonies. These findings align with reports from multiple sources, which identify bee forage shortages, honeybee pests and predators, and agrochemicals as primary challenges across all Ethiopian regions [23–28]. Honeybee pests and predators present a major threat to beekeeping by targeting honeybees and hive products in the study areas. The most common pests and predators include ants, wax moths, honey badgers, bee‐eater birds, small hive beetles, lizards, spiders, death’s head hawkmoths, and wasps. These observations align with reports identifying honeybee pests and predators as key challenges across all regions of Ethiopia [29–32]. Nosemosis affected 60% of hives, higher than prior Ethiopian rates (47%–58%) [31, 32] but lower than in Nigeria (64.29%) or Kenya (83.3%) [33, 34]. Amoeba prevalence reached 83.8%, comparable to Oromia (88%), Amhara (95%), and Benishangul‐Gumuz (60%) [31]. Varroosis hit 82.5%, matching Tigray (82%) [11] but below eastern Amhara (85.9%) and African highs (78.6%–100%) [33–39]. Small hive beetles occurred in 43.8% (above West Hararghe’s 11.7%) [40] and bee lice in 33.8% (between Holeta’s 42% and Wuqro’s 5.5%) [41]. Variations stem from ecology, humidity, management, and bee types.

## 5. Conclusions and Recommendation

The study reveals multiple factors constraining beekeeping in the areas, including bee forage shortages, agrochemical applications, pests and predators, drought and deforestation, diseases, absconding, high costs of improved hives and accessories, and shortages of honeybee colonies. Primary honeybee pests and predators identified in the study areas include ants, wax moths, honey badgers, bee‐eater birds, small hive beetles, lizards, spiders, death’s head hawkmoths, and wasps. Ants, wax moths, and honey badgers proved most destructive, driving colony absconding, weakening, and reduced honey yields. High prevalence of nosemosis, varroosis, amoeba, small hive beetles, and bee lice contributed significantly to honey production losses. Agroecological zones affected amoeba, varroosis, and bee lice occurrences, while hive types influenced nosemosis, amoeba, varroosis, and small hive beetles. The results suggested that beekeepers should prioritize colony strengthening, seasonal management, and regular inspections to control diseases and pests. Further research on seasonal prevalence, economic impacts, long‐term monitoring, and protective technologies is essential.

## Author Contributions

Taye Beyene: conceptualization, methodology, data collection, data analysis, manuscript writing, and laboratory investigation. Mekonen Woldetsadik: conceptualization, methodology, data collection, and laboratory investigation. Desta Abi: conceptualization, methodology, data collection, and laboratory investigation. Sisay Eshetu: conceptualization, data analysis, and manuscript writing.

## Funding

No funding was received for this manuscript.

## Conflicts of Interest

The authors declare no conflicts of interest.

## Data Availability

The data are obtained from the corresponding authors upon reasonable request.
